# Predictors and Effects of Usage of an Online Mindfulness Intervention for Distressed Cancer Patients: Usability Study

**DOI:** 10.2196/17526

**Published:** 2020-10-02

**Authors:** Linda Cillessen, Monique OM van de Ven, Félix R Compen, Else M Bisseling, Marije L van der Lee, Anne EM Speckens

**Affiliations:** 1 Department of Psychiatry Center for Mindfulness Radboud University Medical Center Nijmegen Netherlands; 2 Donders Institute for Brain, Cognition and Behavior Radboud University Nijmegen Netherlands; 3 Department of Medical Psychology Laurentius Hospital Roermond Netherlands; 4 Scientific Research Department Centre for Psycho-Oncology Helen Dowling Institute Bilthoven Netherlands

**Keywords:** internet intervention, eHealth, mindfulness, mindfulness-based cognitive therapy, usage, log data, uptake, adherence, cancer, oncology

## Abstract

**Background:**

One in three cancer patients experience high psychological distress. Mindfulness-based interventions are effective in reducing psychological distress in this patient group. However, these interventions lack availability and flexibility, which may compromise participation in the intervention for cancer patients experiencing late symptoms like fatigue or pain. Therefore, mindfulness-based interventions are increasingly offered via the internet. However, little is known about the usage of these online mindfulness-based interventions.

**Objective:**

The aim of this study was to (1) predict uptake of and adherence to online mindfulness-based cognitive therapy (eMBCT) using baseline patient characteristics (demographic, cancer-related, personality, and psychological variables) and (2) examine the relations between adherence and treatment outcomes in eMBCT for cancer patients.

**Methods:**

A total of 125 cancer patients were assigned to eMBCT in a parent randomized controlled trial comparing MBCT and eMBCT with treatment as usual in distressed cancer patients. Various usage measures of eMBCT were automatically tracked within the online program. Based on activity of use, participants were classified as nonusers, minimal users, low users, and intended users. Questionnaires were used to assess baseline characteristics (preintervention) and outcomes (pre- and postintervention). To answer the research questions, data were analyzed with t tests, χ^2^ tests, and linear regression models.

**Results:**

Based on weekly activity, participants were classified as nonusers (n=17, 13.6%), who completed no exercises in MBCT; minimal users (n=31, 24.8%), who completed at least one exercise of one to three sessions; low users (n=12, 9.6%), who completed at least one exercise of four to seven sessions; and intended users (n=65, 52.0%), who completed at least one exercise of eight to nine sessions. Nonusers had more fear of cancer recurrence at baseline than users (uptake), and intended users were more conscientious than minimal and low users (adherence). Intended users reported a larger reduction in psychological distress and more improvement of positive mental health (ie, emotional, psychological, and social well-being) after the intervention than other participants.

**Conclusions:**

This study showed that adherence was related to improved patient outcomes. Patients with strong fear of recurrence or low levels of conscientiousness should receive extra attention, as they are less likely to respectively start or complete eMBCT. Future research may focus on the development of flexible and adaptive eMBCT programs to fit individual needs.

## Introduction

About one-third of cancer patients and survivors experience high psychological distress due to symptoms of anxiety and depression [1,2]. Mindfulness-based interventions (MBIs) can help to reduce psychological distress in cancer patients, as shown in several large meta-analyses [3,4]. Mindfulness is defined as “paying attention in a particular way: on purpose, in the present moment, and nonjudgmentally” [5]. The two most widely used MBIs (mindfulness-based stress reduction [MBSR] and mindfulness-based cognitive therapy [MBCT]) are usually delivered as group interventions with eight weekly group meetings and a silent day [6,7]. All meetings include meditation exercises (bodyscan, sitting meditations, and gentle movements), psychoeducation, and group discussions. Participants are instructed to practice meditation on a daily basis to cultivate their mindfulness skills.

Despite its beneficial effects, not all distressed cancer patients and survivors are able to participate in a regular face-to-face MBI. As a high proportion of patients have physical problems like severe fatigue [8] or pain [9,10], traveling on a weekly basis to a lengthy meeting at a fixed date and time might be impossible. Therefore, researchers started to investigate whether online MBIs, with their higher accessibility and flexibility, have similar effects as regular face-to-face group MBIs. These online MBIs reduce travel costs and efforts, have 24/7 availability, and may avoid waitlists [11]. Furthermore, participants are able to practice at a location and time they prefer, and at their own pace [12,13].

A recent meta-analysis of online MBIs in various populations showed moderate effects on stress and small effects on depression and anxiety [14]. Focusing on cancer patients, studies on online MBIs revealed promising results. For instance, Zernicke et al [15] found that online mindfulness-based cancer recovery was feasible and reduced symptoms of mood disturbance and stress in a randomized pilot study. In an actively controlled study, online MBCT (eMBCT) reduced fatigue severity in cancer patients [16]. Furthermore, our own trial (the BeMind project) showed that cancer patients reported a moderate reduction in psychological distress after participating in individual eMBCT in comparison with those receiving treatment as usual [17]. Over the course of a 9-month follow-up, eMBCT resulted in an even greater reduction in psychological distress than the classical group-based MBCT [18]. In terms of cost-effectiveness, both treatments were equally cost-effective compared with treatment as usual [19].

Despite growing evidence on the cost-effectiveness of online MBIs, less is known about usage of these online interventions. Usage is relevant to address, as it directly relates to intervention outcomes [20] and can help to optimize interventions [21]. Two relevant aspects of usage are uptake (ie, starting with the intervention) and adherence (ie, receiving an intended dose of the intervention) [22,23]. Uptake and adherence can be tracked with log data from the intervention website. Commonly reported log measures are frequency of use (eg, number of logins), duration of use (eg, duration of each login), and activity (eg, number of completed exercises) [24]. These log measures can be tracked with great objectivity [24], although they only reflect online and not offline engagement with the intervention.

A meta-analysis on predictors of adherence in online interventions among adults found that women demonstrated greater adherence than men. Mixed findings were observed for the relation with age and severity of symptoms [22]. Personality also seems to affect adherence to online interventions. For instance, a study on a web-based occupational health intervention showed that lower levels of negative affectivity and impulsivity and higher levels of alexithymia correlated with less usage of the online intervention [25]. In adults with cancer, uptake was the highest among female patients, while older patients demonstrated a greater adherence to online cognitive behavioral therapy than younger patients [23]. Another study in adults with cancer participating in a web-based cognitive behavioral intervention found that different user groups did not differ in age, education level, or psychological distress [26]. As far as we know, predictors of adherence and its relationship to outcome of online MBIs for cancer patients have not yet been studied.

The aim of this study was to examine the usage of individual eMBCT for distressed cancer patients in relation to outcome. First, we explored whether various baseline patient characteristics (demographic, cancer-related, psychological, and personality variables) could predict uptake and adherence. Second, we tested whether adherence and separate usage measures (number of logins, total time logged in, mean time logged in, number of emails sent to the therapist, and number of assignments completed) were related to treatment outcome in terms of both psychological distress and positive mental health (ie, emotional, psychological, and social well-being). We expected that intended users would gain more from the intervention than low and minimal users.

## Methods

### Study Design

This study concerns secondary analyses of the data from the parent BeMind study, a multicenter randomized controlled trial (RCT) studying the effects of group face-to-face MBCT and eMBCT versus treatment as usual in distressed cancer patients [17,18,27]. This study only includes data of participants immediately randomized to eMBCT or those randomized to eMBCT after treatment as usual. All participants provided written informed consent prior to participation.

### Participants

Distressed cancer patients were recruited through various online and offline media, and were directed to a study website, where they could self-enroll for the study. The inclusion criteria were having any cancer diagnosis; experiencing at least mild psychological distress (a score of ≥11 on the Hospital Anxiety and Depression Scale [HADS] [28,29]); internet access and computer literacy; good command of the Dutch language; and willingness to participate in a mindfulness intervention. The exclusion criteria were severe psychiatric morbidity; change in psychotropic medication within 3 months prior to baseline; and current or previous participation in MBCT or MBSR. More details about the recruitment procedure can be found elsewhere [27].

### Intervention

The online MBCT intervention followed the protocol of Segal et al [30], with some adaptations to fit the needs of the target group, for instance, psychoeducation about grief and cancer-related fatigue. The intervention was designed to be completed in 9 weeks; however, the average time to complete the program was 10.4 weeks (SD 4.0 weeks). Each session was spent on a specific theme, for instance, automatic pilot, communication, or self-care. Participants were provided with information, audio files of guided meditation, and assignments around the theme of the session through a personal secure webpage. The assignments included, for instance, the recording of pleasant or unpleasant events, or how they experienced the meditation exercises. Participants were encouraged to read the information and perform the assigned meditation exercises and assignments within 1 week. The therapist provided feedback on a predetermined day of the week. All therapists had experience in psycho-oncology and were qualified mindfulness trainers according to the criteria of the UK Mindfulness-Based Teacher Network [31]. More details of eMBCT, including screenshots of the intervention, can be found elsewhere [32].

### Measures

#### Log Data

We measured different aspects of usage with log data, which is recommended because different aspects reflect different types of usage [24]. Log data of eMBCT were retrieved from the study website. These included for each login, the time logging in and logging out and the number of assignments saved and submitted. It should be noted that after 30 minutes of inactivity participants were automatically logged out. Furthermore, all emails from participants to therapists were available. From the available data, the following measures were calculated: total time logged in, mean time logged in per login, number of logins (of at least 1 minute), number of completed assignments (which included both those saved by the user and those submitted to the therapist), and number of emails sent to the therapist.

Based on usage, participants were divided into usage groups, which is a common practice in this field [26]. We chose to create categories based on one aspect of activity, namely the amount of sessions in which at least one exercise had been completed, as frequency and duration may be more biased. Regarding frequency, some participants may write their experiences during meditation on paper and add them to the online program at a later moment. Regarding duration, we expected large variations in duration of use, as the ease and speed of writing may differ. Furthermore, activity may be more likely to reflect treatment engagement compared with other usage measures [33]. As eMBCT is a complete program, in which each week provides new knowledge and skills training that builds on the previous week, intended usage was defined as completing at least one exercise of eight to nine sessions. Low usage was defined as completing at least half of the program (ie, four to seven sessions) [34,35]. Minimal users completed at least one exercise of one to three sessions, while nonusers did not complete any of the exercises. When focusing on uptake, nonusers were compared with users (minimal/low/intended), and when focusing on adherence, intended users were compared with low/minimal users.

#### Baseline Characteristics

The following self-reported baseline characteristics were explored as possible predictors: gender, age, education level, cancer type (breast vs other), anticancer treatment intent (curative vs palliative), personality (openness, conscientiousness, extraversion, agreeableness, and neuroticism), baseline psychological distress, positive mental health, rumination, fear of cancer recurrence, and mindfulness skills. Sociodemographic characteristics and cancer-related variables were assessed in the baseline interview and via self-report questionnaires. Psychological predictors included baseline psychological distress (described below), baseline positive mental health (described below), rumination, fear of cancer recurrence, and mindfulness skills. Rumination was measured with the 12-item rumination subscale of the Rumination and Reflection Questionnaire (RRQ) [36]. Fear of cancer recurrence was measured with the nine-item Severity subscale of the Fear of Cancer Recurrence Inventory (FCRI) [37,38]. Mindfulness skills were measured with the Five Facet Mindfulness Questionnaire Short Form (FFMQ-SF), a 24-item self-report questionnaire [39]. Personality was assessed with the NEO Five Factor Inventory (NEO-FFI) [40]. This 60-item self-report questionnaire measures five personality characteristics (openness to experiences, conscientiousness, extraversion, agreeableness, and neuroticism).

#### Outcome Measures

The outcome measures were self-reported psychological distress and positive mental health. Psychological distress was measured with the 14-item HADS (theoretical range 0-42), developed to measure depression and anxiety [28,29]. The HADS has adequate psychometric properties to detect distress in cancer patients [41,42]. Internal consistency in the present study was good (Cronbach α at T_0_=.87). Positive mental health was measured with the Mental Health Continuum Short Form (MHC-SF), a 14-item questionnaire measuring emotional, psychological, and social well-being (theoretical range 0-70) [43]. The MHC-SF has adequate psychometric properties [44]. Internal consistency was excellent (α=.93). Both measures were assessed before and after the intervention.

### Data Analysis

Data were analyzed with SPSS version 22 (IBM Corp). Statistical significance was determined at *P*<.05 (two-sided). Descriptive statistics for participants and usage measures were calculated. With regard to the usage data, 16 participants (12.8%) did not login at all. For these patients, the number of logins was recorded as zero. For the other usage measures, missing values were maintained, as recording them to zero would lead to bias (eg, the mean time logged-in over all participants would be artificially lowered).

Visual inspection of histograms revealed all measures of usage were normally distributed, except for the number of completed assignments, which had a negative skewness, indicating that a large proportion of participants saved all assignments (ceiling effect). For further analyses, the number of completed assignments was dichotomized with median split. A score of one (above the median) indicated that participants saved or submitted either all 58 assignments or all but one assignment (57 assignments), while a score of zero indicated less assignments were completed (<57 assignments). We refer to this variable as completed all assignments (yes/no). Number of logins, total time logged in, mean time logged in, number of emails sent, and number of exercises completed were calculated for each group. In the analyses, the low and minimal user groups were combined to create more equal group sizes.

The first research question focused on prediction of uptake (nonusers vs users) and adherence (intended vs minimal/low users). For each of the user groups, baseline patient characteristics (demographic, cancer-related, personality, and psychological variables) were described. Independent sample *t* tests and *χ*^2^ tests were used to test for significant differences between the user groups regarding uptake or adherence.

To study the relationship between usage and outcome (research question 2), linear regression models were used. These analyses only included participants who actually used the intervention (ie, minimal, low, and intended users). Separate models were run for usage group (minimal/low vs intended), and per usage measure (total time logged in, mean time logged in, number of log-ins, number of assignments completed, and number of emails sent to the therapist) and outcome measure (psychological distress and positive mental health). All models were controlled for baseline levels of outcome measures.

## Results

### Participants

In total, 125 patients with cancer participated in eMBCT. The mean age of the participants was 52 years (SD 10.2). Most participants were female (n=109, 87.2%), had breast cancer (n=76, 60.8%), and were treated with curative intent (n=102, 81.6%). The mean level of psychological distress on the HADS was 17 (SD 6.9).

Regarding the different measures of usage in the total group, participants logged in on average 30.5 times (SD 28.1), with a mean time logged in of 28.1 minutes (SD 19.1) and a total time logged in of 1066 minutes (SD 1217). Participants sent on average nine emails (SD 5.8) and completed most assignments (median 57.5, range 1-58). Seventeen participants (13.6%) were classified as nonusers, 31 (24.8%) as minimal users, 12 (9.6%) as low users, and 65 (52.0%) as intended users. Usage in each of the user groups is displayed in Table 1.

**Table 1:** Descriptive statistics of usage measures in the user groups (N=125).

Variable	Nonusers (n=17)	Users (n=108)		
		Minimal users (n=31)	Low users (n=12)	Intended users (n=65)
Duration: average login time, mean (SD)^a^	3.5 (N/A^b^)^c^	21.4 (17.9)	24.4 (13.1)	32.3 (19.6)
Duration: total login time, mean (SD)^a^	7.0 (N/A)^c^	153.7 (232.5)	588.8 (404.3)	1606.3 (1299.9)
Frequency: number of logins, mean (SD)	0.12 (0.49)	6.6 (6.5)	24.1 (9.4)	51.1 (23.1)
Activity: emails sent	0 (N/A)	3.0 (1.6)	7.7 (4.6)	11.6 (5.3)
Activity: exercises completed^d^	0 (N/A)	8.6 (6.5)	36.8 (7.2)	57.7 (9.2)

^a^Time was measured in minutes.

^b^N/A: not applicable.

^c^One participant in the nonuser group logged in twice, but did not complete any of the exercises, therefore belonging to the nonuser group. The duration scores reflect scores of this participant. Standard deviation cannot be calculated for one score.

^d^As this variable had a strongly skewed distribution, it was dichotomized (median split) for analysis.

### Prediction of Uptake and Adherence

#### Prediction of Uptake

Table 2 presents the baseline characteristics for users and nonusers, and the results of the statistical tests comparing differences between these groups. Nonusers had higher levels of baseline fear of cancer recurrence compared with users (t_118_*=*2.27, *P*=.03). This effect was of a medium to large size (*D*=0.69). There were no other differences between users and nonusers at baseline.

**Table 2:** Descriptive statistics of baseline characteristics of uptake (users vs nonusers) and prediction of uptake assessed with independent sample t tests or χ^2^ tests (N=125).

Characteristic	Nonusers (n=17)	Users (n=108)	Test value (*t* value or *χ*^2^ value) (*df*)	*P* value	Cohen *d*
Age (years), mean (SD)	49.4 (11.4)	52.2 (10.1)	−1.04 (123)	.30	0.26
Gender (female), n (%)	17 (100%)	92 (85%)	2.89 (1)	.09	N/A^a^
Higher education (yes vs no), n (%)	8 (47%)	73 (68%)	2.72 (1)	.10	N/A
Cancer (breast vs other), n (%)	12 (71%)	64 (59%)	0.79 (1)	.37	N/A
Treatment intent (curative vs palliative), n (%)	14 (82%)	88 (82%)	0.01 (1)	.93	N/A
Neuroticism, mean (SD)	38.3 (8.1)	35.8 (7.8)	1.20 (122)	.23	0.31
Extraversion, mean (SD)	37.9 (5.8)	37.9 (6.5)	−0.04 (122)	.97	0.00
Openness, mean (SD)	40.0 (6.0)	40.4 (5.2)	−0.30 (122)	.76	0.07
Altruism, mean (SD)	45.8 (5.6)	46.6 (4.2)	−0.73 (122)	.47	0.16
Conscientiousness, mean (SD)	42.4 (6.8)	41.8 (5.9)	0.40 (122)	.69	0.09
Psychological distress, mean (SD)	16.4 (7.1)	16.8 (6.9)	−0.20 (119)	.84	0.06
Positive mental health, mean (SD)	39.7 (16.0)	37.4 (13.4)	0.59 (119)	.56	0.16
Rumination, mean (SD)	43.2 (9.5)	42.3 (8.3)	0.39 (118)	.70	0.10
Fear of cancer recurrence, mean (SD)	91.5 (18.7)	78.1 (20.3)	2.27 (118)	.03	0.69
Mindfulness, mean (SD)	78.4 (14.4)	77.0 (10.8)	0.44 (119)	.66	0.11

^a^N/A: not applicable.

#### Prediction of Adherence

Table 3 presents the baseline characteristics for low/minimal and intended users, and the results of the statistical tests comparing differences between these groups. Intended users were more conscientious when compared with the combined group of minimal and low users (t_106_=−2.04, *P*=.04). This effect was of a small to medium size (*D*=0.39). There were no other differences between the two groups.

### Relations Between Adherence and Outcomes

Relations between adherence and outcomes are displayed in Table 4. Participants who used the intervention as intended reported less psychological distress (t_86_*=−*2.47, *P*=.02) and more positive mental health after the intervention than minimal and low users (t_86_*=*5.18, *P*=.02). When focusing on specific usage measures, we found that participants who completed all exercises reported less psychological distress (t_86_*=*−2.80, *P*=.01) and more positive mental health (t_86_*=*5.24, *P*=.01) after the intervention than those who did not. Mean time logged in, total time logged in, number of logins, and number of emails sent did not appear to be related to psychological distress or positive mental health after the intervention.

**Table 3:** Descriptive statistics of baseline characteristics of adherence (minimal/low vs intended users) and prediction of adherence assessed with independent sample t tests or χ^2^ tests (N=108).

Characteristic	Minimal/low users (n=43)	Intended users (n=65)	Test value (*t* value or *χ*^2^ value) (*df*)	*P* value	Cohen *d*
Age (years), mean (SD)	52.7 (10.1)	51.8 (10.1)	0.41 (106)	.68	0.09
Gender (female), n (%)	38 (88%)	54 (83%)	0.58 (1)	.45	N/A^a^
Higher education (yes vs no), n (%)	25 (58%)	48 (74%)	2.92 (1)	.09	N/A
Cancer (breast vs other), n (%)	25 (58%)	39 (60%)	0.04 (1)	.85	N/A
Treatment intent (curative vs palliative) n (%)	32 (74%)	56 (86%)	2.36 (1)	.12	N/A
Neuroticism, mean (SD)	36.8 (7.5)	35.1 (8.1)	1.13 (106)	.26	0.22
Extraversion, mean (SD)	36.8 (6.7)	38.7 (6.2)	−1.54 (106)	.13	0.29
Openness, mean (SD)	39.4 (5.8)	41.1 (4.8)	−1.62 (106)	.11	0.32
Altruism, mean (SD)	46.1 (3.7)	47.0 (4.5)	−1.13 (106)	.26	0.22
Conscientiousness, mean (SD)	40.4 (5.8)	42.7 (5.9)	−2.04 (106)	.04	0.39
Psychological distress, mean (SD)	16.6 (6.4)	17.0 (7.4)	−0.31 (105)	.76	0.06
Positive mental health, mean (SD)	37.9 (13.3)	37.1 (13.5)	0.31 (105)	.76	0.06
Rumination, mean (SD)	43.1 (6.7)	41.7 (9.2)	0.88 (105)	.38	0.17
Fear of cancer recurrence, mean (SD)	77.4 (19.7)	78.6 (20.8)	−0.31 (105)	.76	0.06
Mindfulness, mean (SD)	76.1 (9.7)	77.6 (11.6)	−0.69 (105)	.49	0.14

^a^N/A: not applicable.

**Table 4:** Results of linear regression analyses predicting outcomes from usage group (intended vs minimal/low users) and separate usage measures of intervention users (N=89).

Variable^a^		Full model^b^						Predictor							
		Adjusted R^2^		*F* (*df*)		*P*		B		*t* (*df*)		*P*		95% CI	
**Psychological distress**															
	Adherence (minimal/low vs intended users)		0.34		23.76 (2,86)		<.001		−2.87		−2.47 (86)		.02		−5.19 to −0.56
	Number of logins		0.32		20.46 (2,86)		<.001		−0.03		−1.23 (86)		.22		−0.07 to 0.02
	Mean time logged in		0.31		20.69 (2,86)		<.001		−0.04		−1.35 (86)		.18		−0.09 to 0.02
	Total time logged in		0.32		20.40 (2,86)		<.001		−0.001		−1.20 (86)		.23		−0.01 to 0.00
	Emails sent		0.32		19.62 (2,83)		<.001		−0.06		−0.60 (83)		.55		−0.27 to 0.14
	All exercises completed (yes/no)		0.34		24.48 (2,86)		<.001		−2.80		−2.51 (86)		.01		−5.02 to −0.59
**Positive mental health**															
	Adherence (minimal/low vs intended users)		0.49		41.86 (2,86)		<.001		5.18		2.40 (86)		.02		0.88 to 9.48
	Number of logins		0.45		37.28 (2,86)		<.001		0.03		0.90 (86)		.37		−0.04 to 0.11
	Mean time logged in		0.46		36.77 (2,86)		<.001		0.03		0.50 (86)		.62		−0.08 to 0.13
	Total time logged in		0.45		36.64 (2,86)		<.001		0.00		0.33 (86)		.74		−0.01 to 0.01
	Emails sent		0.46		34.77 (2,83)		<.001		0.05		0.28 (83)		.78		−0.32 to 0.43
	All exercises completed (yes/no)		0.51		45.51 (2,86)		<.001		5.24		2.53 (86)		.01		1.12 to 9.36

^a^Of the 108 users, 19 missed either the premeasure or the postmeasure of the outcomes, resulting in a sample size of 89 for these analyses. Three participants missed a score on emails sent, resulting in a sample size of 86 for these analyses.

^b^The full models were controlled for baseline levels of psychological distress or positive mental health.

## Discussion

The aim of this study was to predict uptake and adherence of eMBCT and to examine the association between adherence and treatment outcome of eMBCT in distressed cancer patients. We divided participants into different user groups as follows: nonusers (n=17, 13.6%), minimal users (n=31, 24.8%), low users (n=12, 9.6%), and intended users (n=65, 52.0%). Regarding uptake, nonusers appeared to have more fear of cancer recurrence than users. Regarding adherence, intended users were more conscientious than minimal and low users. Finally, intended users showed a larger reduction in psychological distress and stronger improvement in positive mental health than minimal and low users. We did not find any relations of number of logins, mean and total time logged in, and number of emails sent with treatment outcome. Patients who completed all assignments, however, showed less psychological distress and more positive mental health after the intervention than those who did not.

About half of the participants used eMBCT as intended. A previous study on online cognitive behavioral therapy for cancer patients found a similar percentage of high users (44%) [26].

Regarding prediction of uptake, we found that nonusers had more fear of cancer recurrence than users. It is possible that patients with a high fear of recurrence are more likely to avoid participation, as it means being confronted with their fear. This is in line with a recent study on online self-help for fear of cancer recurrence that found that most patients do not login or express a need for support [45]. As fear of recurrence is an unmet need among cancer patients [46], this group might need more information prior to enrollment in an intervention like eMBCT [47]. A study on an internet-based intervention for depressive symptoms in primary care showed that a brief preparatory informational video increased acceptance of the intervention [48]. A video about eMBCT for distressed cancer patients could discuss evidence for the effectiveness of the intervention, and possible facilitators and barriers (such as confrontation with cancer and fear of recurrence). Preparatory face-to-face conversations with a health care provider prior to eMBCT, in which these topics could be discussed, may also be useful. Involving a partner or a close friend of the patient during eMBCT may help as well [49].

Regarding prediction of adherence, we showed that participants with low conscientiousness have trouble completing eMBCT. This is not surprising, as one of the aspects of conscientiousness is self-discipline [40]. Furthermore, increased conscientiousness is associated with increased mindfulness [50], which might catalyze participation in an online mindfulness program like this. As we previously found that participants with low conscientiousness had better results in eMBCT compared with regular group MBCT [18], more individual feedback by a mindfulness teacher might support them. Alternatively, patients could be offered a preintervention. For example, a study on health behavior showed that participants who were poor in planning benefitted from a preintervention aimed at planning and implementation intentions [51].

Besides fear of cancer recurrence and conscientiousness, we did not find any other predictors of usage of eMBCT. This is in accordance with a recent meta-analysis on predictors of online intervention adherence, which did not find many predictors for adherence either [22]. A proposed behavioral change model for internet interventions suggests that adherence is not only determined by personal characteristics, but also by environmental factors, support, and website characteristics [52], which we also showed in a qualitative study on eMBCT [47]. Future researchers might want to consider improving prediction by including all of these factors. In addition, qualitative methods can deepen our insight into the possible contributors to the usage of eMBCT [47].

Finally, we found that intended users reported less psychological distress and more positive mental health after the intervention than minimal and low users. A similar pattern was found for participants who completed all assignments as compared with those who did not. Thus, it seems that greater activity in eMBCT, rather than the sheer frequency or duration of use, is related to improved treatment outcome. A previous study on an online intervention for depression also found that clinically relevant improvement was related to activity rather than frequency and duration [53]. Our results also match with findings of a meta-analysis on the relation between adherence to homework and treatment outcome in MBIs [21]. The authors concluded that this relation is not linear, suggesting only frequency and duration of practice do not provide a complete picture. Thus, different individual usage patterns may be beneficial (eg, shorter and frequent sessions or longer and less frequent sessions), as long as activity is high.

Regarding clinical implications, our study showed that half of the participants used the program as intended and that higher activity was related to better outcome. As half of the participants did not use the program as intended, participation in these kinds of interventions should be closely monitored. In our case, monitoring took place in the form of weekly written asynchronous contact with a qualified trainer. Possibly, more intensive synchronous digital contact or even blended forms including face-to-face contact might be necessary for particular subgroups of patients [47]. To increase uptake of and adherence to this and similar programs, these programs could be designed as adaptive interventions in which the type (eg, regular group MBCT or eMBCT or hybrid/blended forms), dosage, and even content are individualized and flexible based on patients’ characteristics, preferences, and clinical presentations, as well as their reactions to the intervention [54,55]. However, we should note that although only half of the participants did not use eMBCT as intended, previous results of the BeMind study showed eMBCT to be superior to treatment as usual [17] and, at long-term follow-up, even to regular face-to-face group MBCT [18].

The strengths of this study include comparison of different usage groups in terms of a large number of baseline characteristics and examination of the relationship between usage and treatment outcome. Another strength is that this study was part of an RCT that also included regular group-based MBCT as an intervention arm. Therefore, our sample might be less biased toward participants interested in participating in an online intervention. Some limitations should be mentioned as well. First, the log measures tracked usage of eMBCT. However, participants may have been engaged with the mindfulness training in ways that were not reflected in the eMBCT log measures, as meditation audio files could be downloaded or found on YouTube. Although we assume that usage in eMBCT and engagement with training in other ways highly overlap, we cannot be sure we measured *actual* meditation practice in this study. As meditation is an important aspect of any mindfulness intervention, this is an important point for future research. Second, the amount of emails sent might be a less valid measurement of activity. Participants wrote their personal logs and therapists could reply to those personal logs, creating an additional communication system next to email. Finally, owing to the low number of nonusers, the power to detect possible differences with *t* tests comparing nonusers and users was low, so it is likely that only larger effects were detected.

In conclusion, our study showed that adherence was related to improved patient outcomes. Patients with a strong fear of recurrence or low levels of conscientiousness should receive extra attention, as they are less likely to start or complete eMBCT. Future research may focus on the development of flexible and adaptive eMBCT programs.

## Abbreviations

eMBCT: online mindfulness-based cognitive therapy
HADS: Hospital Anxiety and Depression Scale
MBCT: mindfulness-based cognitive therapy
MBI: mindfulness-based intervention
MBSR: mindfulness-based stress reduction
MHC-SF: Mental Health Continuum Short Form
RCT: randomized controlled trial
